# Basophil Activation Test in the prediction of anaphylactic reactions against Hymenoptera venom in patients suffering from Systemic Mastocytosis

**DOI:** 10.1186/1479-5876-10-S3-P10

**Published:** 2012-11-28

**Authors:** Mark JA Rietveld, Marco WJ Schreurs, Jan AM van Laar, P Martin van Hagen, Paul LA van Daele

**Affiliations:** 1 Dept. of Immunology, Erasmus MC, Rotterdam, the Netherlands; 2 Dept. of Internal Medicine, Erasmus, MC Rotterdam, the Netherlands

## Introduction

Systemic mastocytosis (SM) is a rare disease characterized by proliferation of aberrant mast cells. Mast cells have an important role in anaphylactic reactions. Anaphylaxis against venom of the species Hymenoptera can be a life threatening complication in SM. Unfortunately, it is unpredictable which patients will respond with anaphylaxis after wasp stings.

Mast cells and basophils arise from myeloid cell lineage, but out of different precursor cells. Despite this, these cells are similar in appearance and function. Both cells are stimulated to degranulate by direct triggering, crosslinking of IgE on the cell surface or by the complement system. Different from mast cells, basophils can easily be isolated from peripheral blood. A Basophil Activation Test (BAT) is used to detect and predict anaphylactic reactions against Hymenoptera venom *in vitro*.

## Aim

The aim of this study is to investigate whether a BAT can be used to predict which SM patients will develop anaphylactic reactions for wasps’ venom of the species Hymenoptera.

## Patients and methods

26 SM patients were studied of which 8 had a history of anaphylactic reaction against wasp sting. A BAT was performed with the use of wasp allergen of the species Hymenoptera. The BAT was intended for in vitro determination of expression of CD63 surface marker (a transmembrane protein that fuses with the cellular membrane in degranulation) and CCR3 (a constitutively expressed chemokine for basophil activation) on basophils in EDTA conserved whole blood by flowcytometry upon antigen stimulation in order to evaluate patients’ hypersensitivity for Hymenoptera venom.

## Results

Among the 8 patients with a history of anaphylaxis, we found only one positive BAT for allergen of the species Hymenoptera. In 18 patients without a history of anaphylactic reactions, none had a positive BAT.

## Conclusion

A BAT is not useful for predicting anaphylactic reactions against Hymenoptera venom in patients suffering from SM.

